# Tracking the Expression of Excitatory and Inhibitory Neurotransmission-Related Proteins and Neuroplasticity Markers after Noise Induced Hearing Loss

**DOI:** 10.1371/journal.pone.0033272

**Published:** 2012-03-12

**Authors:** Cherylea J. Browne, John W. Morley, Carl H. Parsons

**Affiliations:** Department of Anatomy and Cell Biology, School of Medicine, The University of Western Sydney, Sydney, New South Wales, Australia; Imperial College London, United Kingdom

## Abstract

Excessive exposure to loud noise can damage the cochlea and create a hearing loss. These pathologies coincide with a range of CNS changes including reorganisation of frequency representation, alterations in the pattern of spontaneous activity and changed expression of excitatory and inhibitory neurotransmitters. Moreover, damage to the cochlea is often accompanied by acoustic disorders such as hyperacusis and tinnitus, suggesting that one or more of these neuronal changes may be involved in these disorders, although the mechanisms remain unknown. We tested the hypothesis that excessive noise exposure increases expression of markers of excitation and plasticity, and decreases expression of inhibitory markers over a 32-day recovery period. Adult rats (n = 25) were monaurally exposed to a loud noise (16 kHz, 1/10^th^ octave band pass (115 dB SPL)) for 1-hour, or left as non-exposed controls (n = 5). Animals were euthanased at either 0, 4, 8, 16 or 32 days following acoustic trauma. We used Western Blots to quantify protein levels of GABA_A_ receptor subunit α1 (GABA_A_α1), Glutamic-Acid Decarboxylase-67 (GAD-67), N-Methyl-D-Aspartate receptor subunit 2A (NR2A), Calbindin (Calb1) and Growth Associated Protein 43 (GAP-43) in the Auditory Cortex (AC), Inferior Colliculus (IC) and Dorsal Cochlear Nucleus (DCN). Compared to sham-exposed controls, noise-exposed animals had significantly (p<0.05): lower levels of GABA_A_α1 in the contralateral AC at day-16 and day-32, lower levels of GAD-67 in the ipsilateral DCN at day-4, lower levels of Calb1 in the ipsilateral DCN at day-0, lower levels of GABA_A_α1 in the ipsilateral AC at day-4 and day-32. GAP-43 was reduced in the ipsilateral AC for the duration of the experiment. These complex fluctuations in protein expression suggests that for at least a month following acoustic trauma the auditory system is adapting to a new pattern of sensory input.

## Introduction

Noise-induced hearing loss (NIHL) is a major and growing problem in the world today. At present, more people are exposed to damaging levels of noise in the workplace, at entertainment venues and the increasing use of personal listening devices (PLD) such as iPods, radios, CD players and mp3 players, is becoming a major concern [1], [2], [3]. It is known that intense noise exposure leads to temporary threshold shifts in hearing, but also that in some instances permanent loss occurs and this may be accompanied by auditory disorders such as tinnitus and hyperacusis in humans [4]. Noise exposure causes a range of changes throughout the auditory pathway including an imbalance of excitatory and inhibitory transmitter systems [5], [6], [7], [8], [9], [10]. This suggests that these molecular changes may be involved in causing auditory disorders, although the mechanisms and site/s of generation have yet to be identified.

This study focuses on the excitatory and inhibitory neurotransmission-related proteins and changes in neuroplasticity-related proteins in the auditory pathway up to one month following acoustic trauma. The central nervous system relies on a fine balance of excitatory and inhibitory inputs [11], and an imbalance may have significant physiological implications in the auditory system. An imbalance may influence the reorganization of tonotopy and could lead to changes in spontaneous activity, which has been previously reported after acoustic trauma [12], [13], [14], [15], [16], [17], [18], [19]. Many studies have reported changes in expression of excitatory and inhibitory neurotransmission-related proteins and the level of neuroplasticity-related proteins in the auditory pathway after acoustic trauma. Changes have been observed in the NMDA receptor subunit 2A (NR2A) mRNA [5], Gamma-Amino-Butyric-Acid (GABA) [7], [8], GABA_A_ receptor alpha 1 subunit (GABA_A_α1) [6], Glutamic-Acid Decarboxylase (GAD-67) [6], [8], Calbindin (Calb1) [20] and GAP-43 [21]. However, the findings of these studies vary significantly from one another, due to the species used, the age of the animals, method of acoustic trauma, method of quantification and assessing protein changes at differing time points.

As mentioned above, many investigations have centered on changes in the central auditory pathway following acoustic exposure. Clearly partial or total hearing loss results in significant changes throughout the auditory pathway that cannot be explained by peripheral loss alone. This statement is supported by the fact that tinnitus persists in patients with acoustic neuroma after transection of the auditory nerve [22], [23]. The Auditory Cortex (AC), Inferior Colliculus (IC) and the Dorsal Cochlear Nucleus (DCN) have each been investigated as sites where hearing related disorders may be generated after acoustic exposure. The AC has been shown to be vulnerable to acoustic exposure through the development of tonotopic reorganisation [15], [16], [18], [24], [25]. The IC has been reported to be functionally and neurochemically altered after acoustic trauma [8], [10], [25]. The DCN has also been implicated as a possible site for the generation of tinnitus-producing signals owing to its tendency to become hyperactive following exposure to tinnitus inducing agents such as intense sound and cisplatin [26].

We have attempted to find the possible site of generation of acoustic disorders. Our investigations centre on the protein changes in the contralateral and ipsilateral AC, IC and DCN within the same animals following acoustic trauma, with the aim of determining which brain regions may be involved in the generation of auditory disorders by assessing the relative changes in protein expression over time. There are also limited investigations into the development of these changes over time, the majority of studies sample time points before and immediately after acoustic exposure, with limited investigations systematically sampling time points after acoustic exposure. Understanding the development of changes that occur after acoustic trauma is of utmost importance for the development of treatments for auditory disorders. Accordingly, we tracked the expression of excitatory and inhibitory neurotransmission-related proteins and the expression of neuroplasticity-related proteins in the contralateral and ipsilateral AC, IC and DCN over 32 days after acoustic trauma. We hypothesized that acoustic trauma would lead to an increase in excitatory and neuroplasticity marker expression and a decrease in inhibitory marker expression in the AC, IC and DCN over time.

## Materials and Methods

### Animals

#### Ethics statement

The experimental procedures were approved by the University of Western Sydney Animal Care and Ethics Committee (ACEC A6670) and conformed to the Australian code of practice for the care and use of animals for scientific purposes – 7^th^ edition. Thirty Long Evans rats aged between 11–14weeks (250 g–350 g) were randomly assigned to 6 groups used to assess the effect of acoustic trauma on the expression of excitatory and inhibitory neurotransmission-related proteins and of neuroplasticity-related proteins in the contralateral and ipsilateral AC, IC and DCN. These groups were a control group (n = 5), day 0 (n = 5), day 4 (n = 5), day 8 (n = 5), day 16 (n = 5) and day 32 (n = 5) post-acoustic trauma group.

### Acoustic Trauma

**Figure 1:**
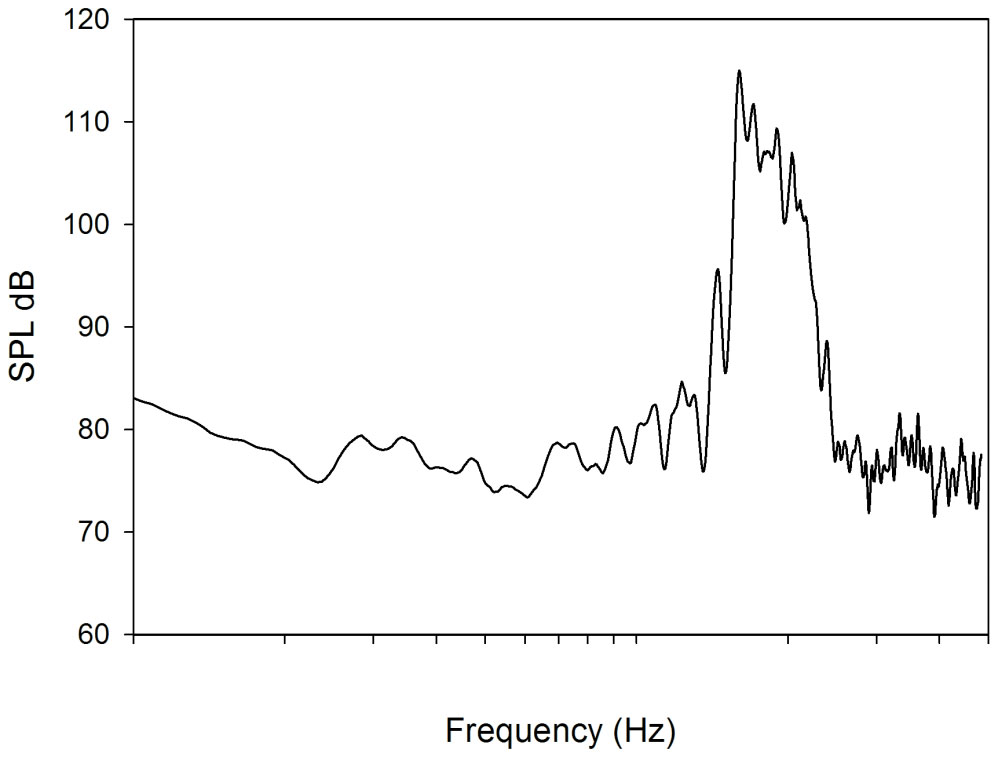
Energy distribution of the noise used in the experiment.

Each rat was anaesthetised via an intraperitoneal (i.p.) injection of ketamine/medetomidine (75 mg/kg;0.5 mg/kg). The animal was placed on a feedback-controlled heating pad with a rectal probe to maintain a constant body temperature of 37°C (Physitemp, Clifton, NJ; Cat #TCAT 2LV Controller). A small tube, 3 mm inner diameter and originating from a speaker (CTS Powerline, Piezo Electric, Woburn, MA) was tightly and securely placed in the animals left ear canal. Rats were unilaterally exposed to a 16 kHz band pass (1/10th octave noise (115 dB SPL)) for 1 hour. The right ear canal remained unblocked during the acoustic trauma. The power spectrum of this stimulus is presented in Figure 1. Control animals were not exposed to the acoustic trauma, but were exposed to a sham acoustic trauma procedure: they underwent the same protocol as the exposed rats, in regards to anaesthesia, ABR testing and were placed in a sound proof room for 1 hour next to the acoustic trauma speaker. The speaker was not turned on during this period. To reverse the effects of the anaesthesia following both procedures, a subcutaneous injection of atipamezole (1 mg/kg) was administered.

### Auditory Brainstem Response Audiograms

Auditory brainstem response (ABR) audiograms were performed before and after acoustic trauma and at 4, 8, 16 and 32 days post acoustic trauma, on both ipsilateral and contralateral ears. The ABR was performed within a sound attenuated room. The animal was anaesthetised as previously described and placed on a heating pad with a rectal probe to maintain a constant body temperature of 37°C. Tucker Davis Technologies (TDT) system 3 Software (OpenEx) and hardware were used to generate stimuli and to acquire data. Stimuli were generated using an RX6 multifunction processor with a sampling rate of 100 kHz. Stimuli were attenuated using TDT PA5 attenuators, and transduced using TDT EC1 electrostatic speakers. The sound was presented to the animal via a small tube, 3.5 mm in diameter, originating from a speaker (TDT EC1), placed gently in the ear canal. Animals were presented with tones at 1, 2, 4, 8, 16 and 32 kHz at a range of 0 dB–85 dB SPL (90 dB was the maximum that the software could deliver). Tone bursts were 3 ms in duration, presented every 17 ms. Each frequency/SPL combination was presented 500 times sequentially from lowest to highest. The resulting evoked potentials were measured using stainless steel electrodes placed subdermally; behind left ear (active), behind right ear (active), in the midline 2.5 cm anterior to the intra-aural axis (reference) and in the rear leg (ground). At the conclusion of the experiment, the electrodes were removed and animals were given a sub-cutaneous (s.c.) injection of atipamezole (1 mg/kg).

### Western Blotting for NR2A, Calb1, GABA_A_Rα1, GAD-67 and GAP-43 protein

Five animals from each of the six exposure groups and their respective controls (30 in total) were decapitated at 0, 4, 8, 16 or 32 days post acoustic trauma and their brains snap frozen in liquid nitrogen and stored at −80°C until processed. At the time of the experiment, the brains were semi-thawed in 3 ml of protease inhibitor cocktail (Sigma-Aldrich Inc. Cat #8340) and the contralateral and ipsilateral AC, IC and DCN were removed. Regions were identified in accordance with a rat atlas [27]. Each tissue sample was placed in 500 µl of protease inhibitor cocktail. All tissue samples were kept on ice (4°C) throughout the experiment unless otherwise specified. Tissues were homogenised with protease inhibitor cocktail with a hand held tissue grinder (Pellet Pestle Motor, Kontes, Vineland, NJ). The homogenate was passed through a 27G syringe needle to further shear the tissue, then was centrifuged for 10 minutes at 1000 rpm and the supernatant was collected. The supernatant was then centrifuged for 20 minutes at 12000 rpm and the pellet was collected. The pellet was resuspended in protease inhibitor cocktail; 100 µL (AC and IC) and 50 µL (DCN), and passed through a 30G syringe needle. Five µl was removed from each sample for a protein assay (Bio-Rad, Hercules, CA). Each sample received 2× sample buffer; 100 µL (AC and IC) and 50 µL (DCN). Forty µg of protein was loaded in each well and samples were run for 30 minutes at 60 volts and 90 minutes at 120 volts on 10% SDS-PAGE gel. The gel was transferred overnight at 30 mA for a minimum of 17 hours. The gel was transferred onto 0.2 µm pore size nitrocellulose membrane (Sigma-Aldrich, St Louis, MO). The resulting membranes were blocked with 5% skim milk on a rotator for 1 hour. The membrane was probed with a primary antibody multiplex diluted in 3% skim milk for 2 hours, which consisted of GABA_A_Rα1 (Chemicon, Temecula, CA; Cat #06-868) at a concentration of 1∶4000, GAD-67 (Santa Cruz Biotechnologies; Cat #28376) at a concentration of 1∶400, NR2A (Abcam, Cambridge, MA; Cat #14596-50) at a concentration of 1∶3000, Calb1 (Abcam, Cambridge, MA; Cat #9481-500) at a concentration of 1∶150 and GAP-43 (Abcam, Cambridge, MA; Cat #50608) at a concentration of 1∶400. The membrane was washed repeatedly and probed with secondary antibodies, goat anti rabbit (Sigma-Aldrich, St Louis, MO; Cat #4914) at a concentration of 1∶1000 and goat anti mouse (Sigma-Aldrich, St Louis, MO; Cat #4416) at a concentration of 1∶750 for 1 hour. The membrane was incubated for 5 minutes with chemiluminescent substrate, Supersignal West Pico (Pierce, Rockford, IL; Cat #34087) and Supersignal West Femto (Pierce, Rockford, IL; Cat #34094) when the signal was low. The membranes were placed on CL exposure films (Pierce, Rockford, IL; Cat #34090) for varying times depending on the signal. The films were processed in an x-ray film processor (Agfa CP-1000). The films were scanned in a LAS-4000 imaging system (Fujifilm Life Science, USA) and images were digitised. The images were analysed using Multi-Gauge Analysis Software Version 2.0 (Fujifilm Life Science, USA), which quantifies the level of density in each band selected compared to the background. Deep Purple Total Protein Stain (GE Healthcare Life Sciences; Cat #RPN6305) was used to standardise results.

### Statistical analysis

Statistical analyses were performed using SPSS (IBM Corporation, Somers, NY). The pre and post ABR audiograms (specifically the SPLs) of the groups were compared at individual frequencies, but not across frequencies, using a two tailed student's t-test with a P value of <0.05 considered significant. Control and post acoustic trauma ABR threshold shifts were compared at individual frequencies, but not across frequencies, using a one-way ANOVA and Tukey's post-hoc multiple comparison test with a P value of <0.05 considered significant. If no response was evoked at a SPL of 85 dB, we assigned a SPL threshold of 90 dB. For multiple comparisons of the western blot data, a one-way ANOVA and Tukey's post-hoc multiple comparison test were used with a P value of <0.05 considered significant.

## Results

### Auditory Brainstem Response Audiograms

**Figure 2:**
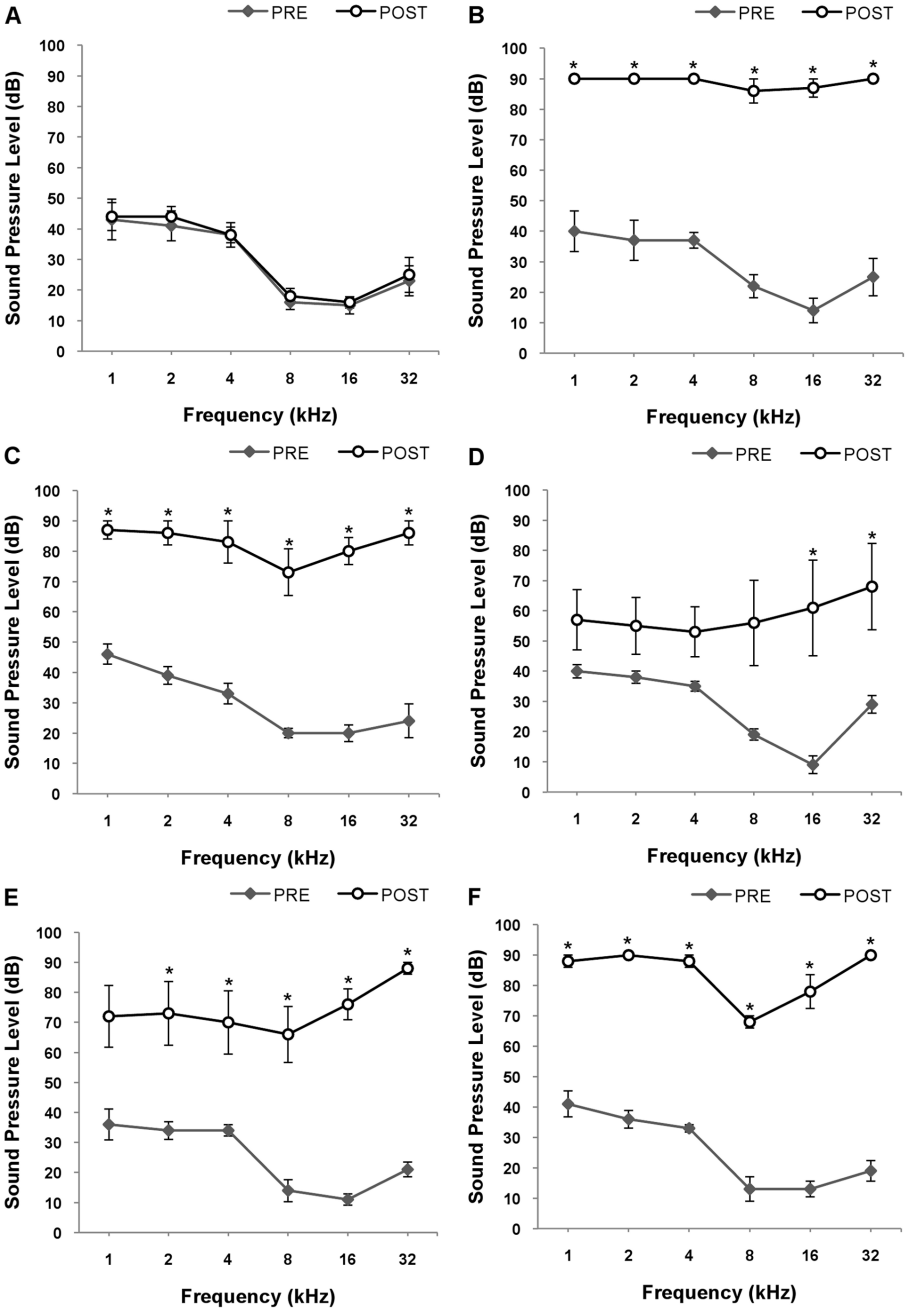
ABR Audiograms of absolute thresholds. Pre-lesion recordings are compared to (A) post-lesion (SHAM); (B) 0 days post-lesion; (C) 4 days post-lesion; (D) 8 days post-lesion; (E) 16 days post-lesion and (F) 32 days post-lesion recordings. Results are presented as the mean ± SEM of SPL (dB). *p<0.05.

ABR audiograms indicate significant threshold shifts following acoustic trauma. Figure 2A–F presents the mean ABR audiograms of all animals obtained prior to the acoustic trauma and at 0 (Fig. 2B), 4 (Fig. 2C), 8 (Fig. 2D), 16 (Fig. 2E) and 32 (Fig. 2F) days post acoustic trauma. All values in the figures are expressed as mean ± SEM. The control sham trauma group showed no significant difference across all frequencies (Fig. 2A), which was expected as the animals received no acoustic trauma. The 0 (Fig. 2B), 4 (Fig. 2C), 8 (Fig. 2D), 16 (Fig. 2E) and 32 (Fig. 2F) day groups showed a significant threshold increase across most frequencies. The lesioned (ipsilateral) ear indicated significant threshold shifts after acoustic trauma. These increases were sustained up to 4 days. At day 8, the high frequencies remained elevated (16 kHz and 32 kHz), whilst the lower frequencies returned to normal levels. At 16 days, these threshold shifts increased again, except for 1 kHz which remained at normal. At 32 days, threshold shifts across all frequencies were significantly increased. Table 1 presents the threshold shifts in dB SPL/frequency/group with p values. The ABR audiograms of the contralateral ear are presented in Table 2 and indicate no significant changes in threshold shifts.

**Table 1:** Post Acoustic Trauma Threshold shifts in all exposure groups (Ipsilateral Ear).

	Frequency					
Group	1 kHz	2 kHz	4 kHz	8 kHz	16 kHz	24 kHz
**Control (SHAM)**	1 dB (p = 0.9)	3 dB (p = 0.6)	0 dB (p = 1.0)	2 dB (p = 0.6)	1 dB (p = 0.8)	2 dB (p = 0.8)
**Day 0**	50 dB **(p = 0.003)**	53 dB **(p = 0.002)**	53 dB **(p<0.001)**	64 dB **(p<0.001)**	73 dB **(p<0.001)**	65 dB **(p<0.001)**
**Day 4**	41 dB **(p = 0.019)**	47 dB **(p = 0.007)**	50 dB **(p = 0.001)**	53 dB **(p = 0.004)**	60 dB **(p = 0.001)**	62 dB **(p<0.001)**
**Day 8**	17 dB (p = 0.723)	17 dB (p = 0.807)	18 dB (p = 0.491)	37 dB (p = 0.076)	52 dB **(p = 0.003)**	39 dB **(p = 0.018)**
**Day 16**	36 dB (p = 052)	39 dB **(p = 0.038)**	36 dB **(p = 0.017)**	52 dB **(p = 0.005)**	65 dB **(p<0.001)**	67 dB **(p<0.001)**
**Day 32**	47 dB **(p = 0.006)**	54 dB **(p = 0.002)**	55 dB **(p<0.001)**	55 dB **(p = 0.002)**	65 dB **(p<0.001)**	71 dB **(p<0.001)**

Significant p values in bold.

**Table 2:** Post Acoustic Trauma Threshold shifts in all exposure groups (Contralateral Ear).

	Frequency					
Group	1 kHz	2 kHz	4 kHz	8 kHz	16 kHz	24 kHz
**Control (SHAM)**	5 dB (p = 0.8)	2 dB (p = 0.6)	2 dB (p = 1.0)	2 dB (p = 0.6)	6 dB (p = 0.8)	1 dB (p = 1.0)
**Day 0**	−3 dB (p = 0.8)	−1 dB (p = 1.0)	3 dB (p = 1.0)	3 dB (p = 1.0)	2 dB (p = 0.9)	0 dB (p = 1.0)
**Day 4**	−4 dB (p = 0.7)	−3 dB (p = 0.9)	−7 dB (p = 0.5)	−5 dB (p = 0.6)	−3 dB (p = 0.3)	−3 dB (p = 1.0)
**Day 8**	−13 dB (p = 0.8)	9 dB (p = 0.8)	3 dB (p = 1.0)	1 dB (p = 1.0)	7 dB (p = 1.0)	−2 dB (p = 1.0)
**Day 16**	−1 dB (p = 1.0)	0 dB (p = 1.0)	1 dB (p = 1.0)	1 dB (p = 1.0)	2 dB (p = 0.9)	−1 dB (p = 1.0)
**Day 32**	−4 dB (p = 0.7)	−4 dB (p = 0.9)	0 dB (p = 1.0)	−2 dB (p = 0.9)	−1 dB (p = 0.5)	−3 dB (p = 1.0)

Significant p values in bold.

### Protein Expression in dominant Auditory Pathway (Contralateral AC, Contralateral IC and Ipsilateral DCN)

**Figure 3:**
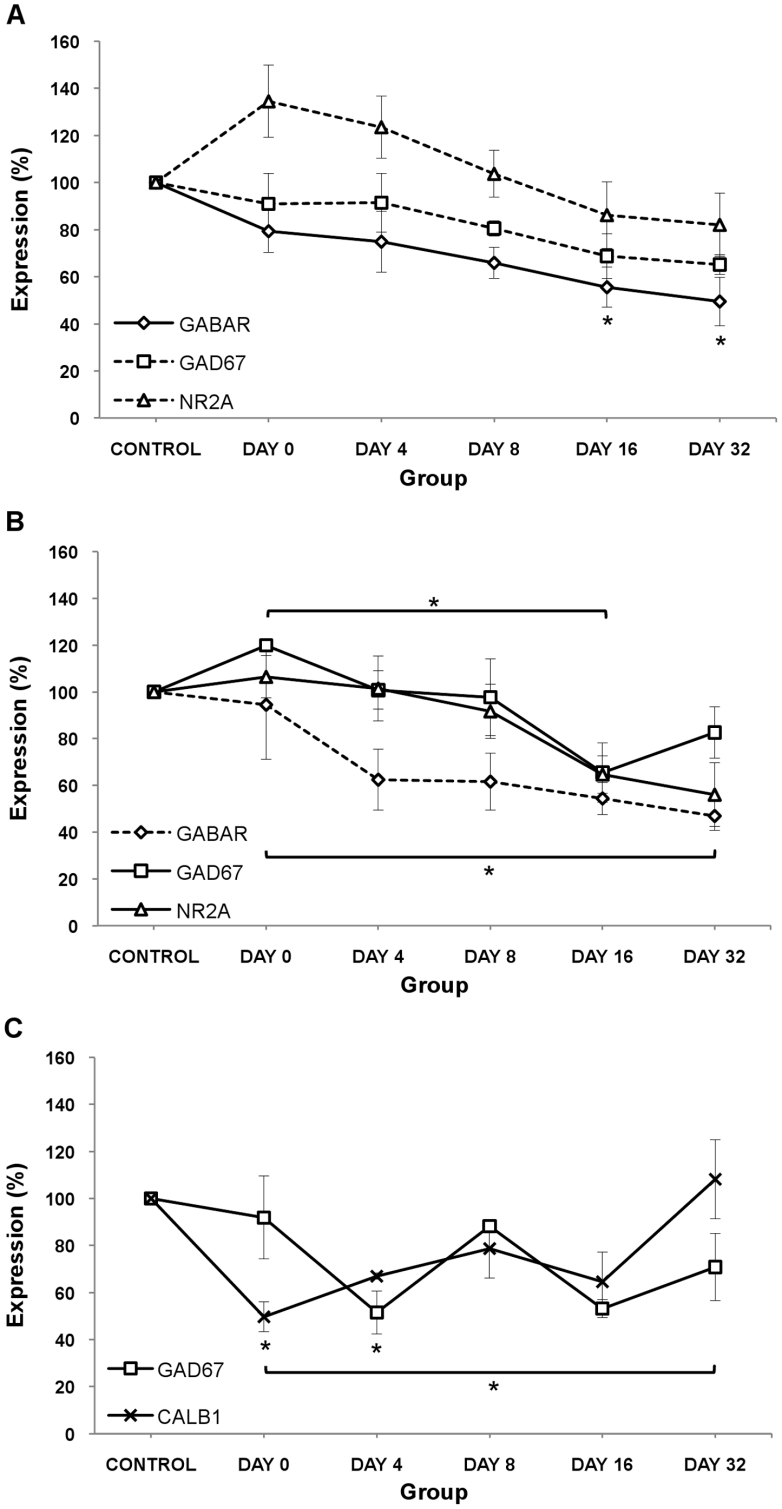
Expression of proteins of interest after acoustic trauma in Dominant Auditory Pathway. (A) Contralateral AC; (B) Contralateral IC and (C) Ipsilateral DCN. Results presented are the mean ± SEM of density units expressed as a % compared to controls. NOTE: only protein expression that reached statistical significance (full lines) or indicates trends (dotted lines) are plotted on the graphs. * p<0.05.

Figure 3 shows the pattern of protein expression in contralateral AC, contralateral IC and ipsilateral DCN, which is the dominant auditory pathway with respect to the exposed ear. Tukey's post-hoc analysis showed statistically significant changes in the dominant auditory pathway compared with the control animals and between groups, and these are summarized in the following sections. GAP-43 protein did not show any significant changes in any of the groups over time.

### Excitatory neurotransmission related proteins

In the contralateral IC, NR2A showed a 44% decrease when comparing day 0 to day 32 (p<0.01) (Fig. 3B). In the ipsilateral DCN, Calb1 showed a 50% decrease at day 0 (p<0.05), compared to controls, and showed a 118% increase when comparing day 0 to day 32 (p<0.05) (Fig. 3C).

### Inhibitory neurotransmission related proteins

In the contralateral AC, GABA_A_α1 showed a 44% decrease at day 16 (p<0.05) and a 51% decrease at day 32 (p<0.01), compared to controls (Fig. 3A). In the contralateral IC, GAD-67 showed a 45% decrease when comparing day 0 to day 16 (p<0.05) (Fig. 3B). In the ipsilateral DCN, GAD-67 showed a 49% decrease at day 4 (p<0.05), compared to controls (Fig. 3C).

### Protein Expression in non-dominant Auditory Pathway (Ipsilateral AC, Ipsilateral IC and Contralateral DCN)

**Figure 4:**
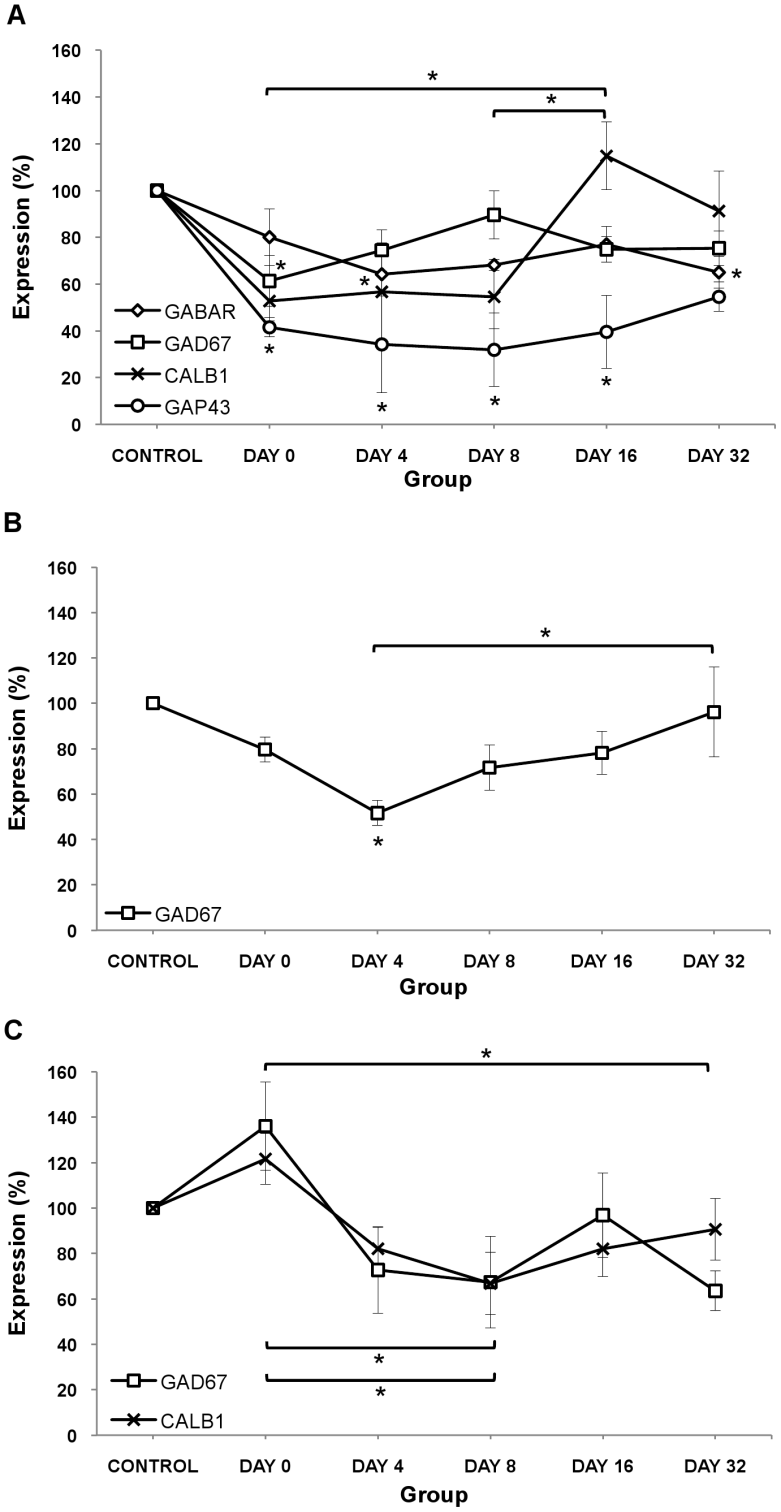
Expression of proteins of interest after acoustic trauma in Non-dominant Auditory Pathway. (A) Ipsilateral AC; (B) Ipsilateral IC and (C) Contralateral DCN. Results presented are the mean ± SEM of density units expressed as a % compared to controls. NOTE: only protein expression that reached statistical significance are plotted on the graphs. * p<0.05.

Figure 4 shows the pattern of protein expression in ipsilateral AC, ipsilateral IC and contralateral DCN, which is the non-dominant auditory pathway with respect to the exposed ear. Tukey's post-hoc analysis showed statistically significant changes in the dominant auditory pathway compared to the control animals and between groups, and these are summarized as follows.

### Excitatory neurotransmission related proteins

In the ipsilateral AC, Calb1 showed a 117% increase when comparing day 0 to day 16 (p<0.05) (Fig. 4A). In the contralateral DCN, Calb1 showed a 45% decrease when comparing day 0 to day 8 (p<0.05) (Fig. 4C). NR2A protein did not show any significant changes in any of the groups.

### Inhibitory neurotransmission related proteins

In the ipsilateral AC, GABA_A_α1 showed a 19% decrease at day 4 (p<0.05) and a 35% decrease at day 32 (p<0.05) compared to controls, GAD-67 showed a 39% decrease at day 0 (p<0.05) compared to controls (Fig. 4A). In the ipsilateral IC, GAD-67 showed a 48% decrease at day 4 (p<0.05) compared to controls and an 86% increase when comparing day 4 to day 32 (p<0.05) (Fig. 4B). In the contralateral DCN, GAD-67 showed a 50% decrease when comparing day 0 to day 8 (p<0.05) and a 53% decrease when comparing day 0 to day 32 (p<0.05) (Fig. 4C).

### Neuroplasticity related proteins

In the ipsilateral AC, GAP-43 showed a 58–68% decrease when comparing day 0 (p<0.05), day 4 (p<0.01), day 8 (p<0.01) and day 16 (p<0.05) to controls (Fig. 4A).

## Discussion

We have quantified the protein expression of GABA_A_Rα1, GAD-67, NR2A, Calb1 and GAP-43 in the ipsilateral and contralateral AC, IC and DCN of the young adult rat up to 32 days following acoustic trauma. We have demonstrated that acoustic trauma significantly alters the normal levels of excitatory and inhibitory neurotransmission-related proteins and neuroplasticity-related proteins in the auditory pathway of the rat over time. Our findings suggest that these changes in protein expression may serve as part of the molecular mechanism underlying significant physiological changes which lead to acoustic disorders following acoustic trauma.

### The Effect of Acoustic Trauma on Hearing Thresholds

The degree of hearing loss produced by exposure to a sound is dependent on the loudness of the sound and the duration of the exposure. Temporary Threshold Shifts (TTS), which are temporary shifts in hearing thresholds, may be observed immediately following exposure [28], [29], [30], and if the sound is loud enough it may produce permanent elevations in thresholds resulting in permanent threshold shifts (PTS) [31].

The hearing loss produced by our stimulus can be characterized by significant threshold shifts across all frequencies and all time points. The threshold shifts ranged from 49–74 dB at Day 0, 46–67 dB at Day 4, 18–48 dB at Day 8, 31–65 dB at Day 16 and 47–67 dB at Day 32. It is interesting to note the gradual reduction of hearing thresholds over the first eight days. Day 8 suggests that some hearing function has recovered after the acoustic trauma. However, this recovery is not sustained and hearing thresholds begin to increase.

The acoustic trauma we used caused a significant increase in hearing thresholds for the duration of our study. Others who have used a similar acoustic trauma treatment have shown mixed results. Turner [32], using the same strain of rats reported that after 4 months recovery thresholds remained elevated for all frequencies tested (except 10 kHz and 32 kHz). Additionally, Bauer [29] reported that threshold shifts remained elevated 7 months after being exposed to a 16 kHz octave band noise at 105 dB for 1 hour. In contrast, Wang and Caspary [33] using Fisher Brown Norway rats showed full recovery by 16 weeks post-exposure. It should be noted that the maximum hearing loss in the present study occurred at 16 kHz and 32 kHz and at those frequencies the losses were greater than those of comparable studies [29], [32], [33].

The initial threshold shifts may be a result of peripheral damage to the auditory system. The immediate changes that occur in inner hair cells (IHC) and outer hair cells (OHC) of the cochlea are quite variable (see [34] for extensive review). It is known that IHC and OHC cannot regenerate in humans and most mammals [35], The observation of the Day 8 recovery may suggest a combination of central and peripheral events playing a role in the temporary recovery of hearing function after acoustic trauma. It is not in disagreement with findings which suggest that peripheral recovery can occur, followed by central degenerative changes [36], [37] or changes in excitatory transmission [38].

### Protein Expression

Western blot data demonstrated that all proteins of interest were expressed in the contralateral and ipsilateral AC, IC and DCN of the adult Long Evans rat. Fluctuations in the expression of excitatory and inhibitory neurotransmission-related proteins may play a role in excitotoxicity, decreased neuronal survival and the overall decrease of inhibitory neurotransmission. A significant decrease in neuroplasticity-related proteins may play a role in reorganization of tonotopic maps, which has been shown to occur after acoustic trauma [16], [18], [19], [25]. These changes could ultimately lead to the development of acoustic disorders, such as tinnitus and hyperacusis.

### The Effect of Acoustic Trauma on Excitatory Neurotransmission-Related Proteins

We found that the expression of the calcium binding protein (CBP) Calb1 decreased immediately after acoustic trauma in the ipsilateral DCN, followed by a recovery to normal level over the 32 days. Calb1 decreased slightly in the contralateral AC followed by recovery at Day 16. It has been postulated that Calb1 has a neuroprotective effect against excitotoxic damage caused by disruption of intracellular calcium homeostasis [39]. A possible reason for the decrease in Calb1 could be that the production of Calb1 is disrupted and therefore could be responsible for excitotoxic damage that may occur in the auditory system after acoustic trauma. Excitotoxicity in the cochlea and central auditory pathway has been researched extensively and is postulated to be a major contributor to the development of acoustic disorders [38], [40]. Indeed, Muly and colleagues have reported evidence of excitotoxic damage following unilateral acoustic trauma in the chinchilla [38]. In excitotoxicity, elevations of Ca^2+^ progress to an irreversible loss of Ca^2+^ homeostasis and neuronal death. In this study, a reduction of Calb1 in the ipsilateral AC and DCN could suggest that a dysfunction has occurred in the production of Calb1 which would affect its function as a cytoplasmic calcium buffer, thereby no longer being able to protect cells against excitotoxic damage. However, it is known that there are other CBPs such as Calretinin and Parvalbumin, which carry out similar functions to Calb1 [41]. Thus it would be important to investigate the changes of other CBP after acoustic trauma as Calb1 levels were not increased. It has been shown that Calretinin and Parvalbumin protein levels increase in mice that were exposed to repeated noise trauma [42]. The immediate decrease of Calb1 in the ipsilateral DCN and the contralateral AC that we observed has not been shown in other studies. The expression of Calb1 in the contralateral DCN slightly increased immediately but returned to normal levels over time. These contralateral DCN findings support previous studies which have found that Calb1 increases in the DCN immediately after acoustic trauma [43], [44].

The other excitatory neurotransmission-related protein that we investigated was NR2A. The expression of NR2A protein had a tendency to decrease over the month following the trauma in the contralateral IC. NMDA receptors containing the NR2A subunit display fast kinetics and NR2A is the most widespread NMDA receptor subunit in the mature brain [45]. This decrease could suggest that the auditory system attempts to compensate for increased excitation; the down regulation of the receptors may serve to reduce increased levels of excitation. Kim and colleagues showed an increase in glutamate release after acoustic trauma, followed by a decrease and a subsequent increase [37]. Our results support previous research by Marionowski et al, which found a decrease in the expression of NR2A mRNA after ototoxic exposure in IC and CN of young rats [5].

A decrease in the expression of NR2A in the contralateral IC could also suggest a reduction in neuronal survival and subsequently neuronal death, because injury induced activation of NR2A containing NMDARs functions as a pro-survival signal [46]. A decrease in neuronal survival after acoustic trauma has been shown in numerous studies. An increase of apoptotic markers was observed in the CN after severe acoustic trauma [47]. Other studies have observed an increased in cell death and degeneration after acoustic trauma in the CN [48], [49], IC [49] MGB and AC [49], [50].

The fluctuations we observed in the protein levels of the CBP, Calb1, suggest that there may be fluctuations of excitation in central auditory structures after acoustic trauma. The slight decreases we observed in the protein levels of the NMDA receptor subunit NR2A suggests that they may not have a direct effect on excitation changes, but may have an effect on neuronal survival of auditory neurons.

### The Effect of Acoustic Trauma on *Inhibitory Neurotransmission-Related Proteins*

This is the first study that has investigated the effect of acoustic trauma on the expression of GABA_A_α1 in the ipsilateral and contralateral AC. After acoustic trauma, the expression of GABA_A_α1 decreased over time firstly in the ipsilateral AC and followed by the contralateral AC. Previous studies have shown that the expression of GABA_A_α1 also decreases in other regions of the auditory pathway, such as the IC and DCN [6], [14]. This down-regulation could result in a significant decrease in inhibition in these auditory areas on both ipsilateral and contralateral sides. A consequence of the reduction of inhibitory processes is that increased excitation may occur in the AC after acoustic trauma, resulting in an altered pattern of spontaneous activity in the AC. Indeed, changes in spontaneous activity and neuronal excitability has been observed across the auditory pathway after acoustic trauma, specifically in DCN [26], the IC [6], [14], [17] and the AC [12], [19].

After unilateral acoustic trauma, the expression of GAD-67 increased slightly in the contralateral IC and DCN, followed by a recovery to normal levels over time. A decrease was observed immediately in the ipsilateral AC, within the first week in the ipsilateral IC but the levels of GAD-67 in both regions returned to normal over time. Slight fluctuations were observed in the contralateral DCN followed by a recovery to normal levels over time. GAD-67 is an enzyme that converts intracellular glutamate into the neurotransmitter GABA [51]. A decreased expression of GAD-67 will lead to reduced levels of GABA, which has been previously observed after acoustic trauma [8], [52], and can ultimately lead to an overall decrease in inhibition. The changes of GAD-67 were the most widespread finding in this study and suggest that a disruption in inhibitory transmission plays an important role in the development of acoustic disorders.

The decreases we observed in the protein levels of the GABA_A_α1 receptor subunit and the GABA synthesis enzyme GAD-67 supports the theory of Dong et al., implying that both pre- and post-synaptic mechanisms contribute to deafness- related loss of inhibition [53]. The possibility that presynaptic GABA synthesis may diminish in the auditory pathway after ipsilateral partial deafness is supported by a study showing a loss of GABA positive neurons in the CN of aged rats with hearing loss [52] and decreased GABA release in the IC [8]. It has been demonstrated that GABA release and binding, as well as GAD-67 expression are down-regulated in the IC after hearing loss [54], [55], [56]. The down-regulation of GABA_A_α1 and GAD-67 would most likely result in an overall decrease in neuronal inhibition in the auditory pathway. This could lead to increases in spontaneous activity, neuronal synchrony and excitability, which has been observed across the auditory pathway after acoustic trauma [13], [17], [19], [57]. Prolonged synchronous firing has been observed in the AC after acoustic trauma [12], [19], which can lead to use-dependent synaptic modifications, Long Term Potentiation (LTP). LTP is thought to promote the increase of synaptic strength and this may lead to the development of auditory disorders such as tinnitus [58].

### Neuroplasticity-related proteins

The expression of GAP-43 has been shown to increase during heightened levels of neuroplasticity, as it is located in the growth cones of neurons [59]. Previous research indicates that GAP-43 also plays a key role in axonal guidance [60], [61], GAP-43 (−/−) mice show defects in path finding of retinal ganglion cell axons across the optic chiasm [62]. Over expression of GAP-43 leads to formation of new synapses and enhanced sprouting after injury whereas reduced sprouting is found with a mutant GAP-43 that cannot be phosphorylated by Protein Kinase C. This control of sprouting implies that GAP-43 is an intrinsic determinant of the neurons growth state. We observed a significant decrease in the overall expression of GAP-43 in the ipsilateral AC. This may have an impact on anatomical reorganization of tonotopic maps after NIHL, as previous studies suggest that reduced GAP-43 expression may alter the fine tuning of a cortical map through a combination of path finding and synaptic plasticity mechanisms [63].

### Significance of Time-Course Changes

To date, there are limited investigations into the time-course of changes that occur in the auditory pathway after acoustic trauma. This is the first study that has investigated changes in the ipsilateral and contralateral AC, IC and DCN in the same animals over time.

Samples were taken as early as 30 minutes after noise trauma (day 0 time point). At this time point we observed significant changes in the expression of Calb1; in the ipsilateral DCN, GAD-67 and GAP-43; in the ipsilateral AC. This observation indicates that these proteins act immediately after the acoustic trauma and may suggest a role in an immediate active fine-tuning process.

Significant changes in both contralateral and ipsilateral DCN involved GAD-67 and Calb1. Over the time-course, GAD-67 and Calb1 were very closely related, both experiencing fluctuations at similar time points. As Calb1 is an indicator of excitation and GAD-67 is involved in synthesizing GABA, it may suggest that there is a connection between these proteins that acts to balance excitatory and inhibitory actions in the DCN normally and after an acoustic insult. These fluctuations over time may indicate that the nervous system continually attempts to rebalance the changes that are occurring in response to altered input. For example when the Calb1 increased, GAD-67 increased in order to produce more GABA, and when Calb1 decreased, GAD-67 also decreases due to the decreased demand for GABA. A previous study has shown that neurons in the olfactory bulbs of mice co-express Calb1 and GAD-67 [64], however there is no study that has looked at the co-expression of these two proteins over time. It is not known the extent to which the levels of Calb1 and GAD-67 relative to each other over time.

The contralateral AC and IC show similar significant decreases and near-significant trends in the levels of NR2A, GAD-67 and GABA_A_α1. This may indicate the AC and IC contralateral to the lesioned ear respond in a similar fashion. The overall potential of the cells would be excitatory which as previously discussed can lead to LTP and may facilitate synaptic plasticity. It would be beneficial to research into the tonotopic changes of the AC and IC to identify whether these protein changes correlate to cortical reorganization and increases of spontaneous activity over time.

The ipsilateral IC experienced a decrease in the expression of GAD-67 within the first week after acoustic trauma. The decrease in the GAD-67 may indicate a malfunction, which would lead to decreased levels of GABA which would result in increased excitation. However, there were no significant changes in the expression of excitatory neurotransmission related proteins in the ipsilateral IC across the time-course. There have been no other reports of the ipsilateral IC changing significantly after acoustic trauma.

Many significant changes were observed in the ipsilateral AC. GAD-67 and GABA_A_Rα1 protein levels were observed to be closely related over the time-course. This was expected as both of these proteins work together in maintaining the balance of excitation and inhibition. We observed fluctuations of Calb1 over the time-course which may indicate that excitation fluctuations were. The decrease GAP-43 may suggest that the ipsilateral AC experiences maladaptive plastic changes, due to the neuronal path finding properties of GAP-43.

In summary, we provide the first 32 day time-course investigation of protein expression of NR2A, Calb1, GABA_A_Rα1, GAD-67 and GAP-43 in the contralateral and ipsilateral AC, IC and DCN utilizing a rat model of unilateral NIHL. We have investigated the development of these protein changes, in order to gain a deeper understanding of the underlying mechanisms of NIHL. Gaining more knowledge about the underlying mechanisms of NIHL is crucial for the development of therapeutic intervention.

Our findings provide evidence that acoustic trauma significantly affects the balance of excitatory and inhibitory neurotransmission-related proteins and neuroplasticity-related proteins in the rat auditory pathway. Our results suggest that the characteristic decrease of inhibition that previous researchers have observed after acoustic trauma, may not necessarily be due to significant increases of excitatory neurotransmission-related proteins; rather a significant *decrease* of inhibitory-neurotransmission related proteins which leads to an overall increase in excitation. This does not support our original hypothesis.

In addition to confirming changes in excitatory and inhibitory-neurotransmission related protein expression, we have shown for the first time that mechanisms from the contralateral auditory pathway may play a role in functional compensation after NIHL. Changes in the ipsilateral and contralateral AC, IC and DCN, may reflect an attempt to balance excitatory and inhibitory transmission following noise-induced hearing loss. The resulting imbalances may contribute to the generation of acoustic disorders.
